# Peripapillary hyper-reflective ovoid mass-like structures (PHOMS) in AQP4-IgG-positive neuromyelitis optica spectrum disease (NMOSD) and MOG-IgG-associated disease (MOGAD)

**DOI:** 10.1007/s00415-022-11381-8

**Published:** 2022-10-16

**Authors:** Jonathan A. Gernert, Rebecca Wicklein, Bernhard Hemmer, Tania Kümpfel, Benjamin Knier, Joachim Havla

**Affiliations:** 1 Institute of Clinical Neuroimmunology, LMU Hospital, Ludwig-Maximilians University Munich, Munich, Germany; 2 Department of Neurology, Klinikum rechts der Isar, Technical University of Munich, Munich, Germany; 3 Data Integration for Future Medicine (DIFUTURE) Consortium, Munich, Germany; 4 Munich Cluster for Systems Neurology (SyNergy), Munich, Germany

**Keywords:** PHOMS, NMOSD, MOGAD, Demyelinating disease, OCT

## Abstract

**Background:**

Peripapillary hyperreflective ovoid mass-like structures (PHOMS) have recently been described as new optical coherence tomography (OCT) marker. It is not yet clear whether the occurrence of PHOMS is disease-specific or disease-spanning. PHOMS have been described in 16–18% of patients with multiple sclerosis (MS). Currently, no data on the prevalence of PHOMS in other demyelinating diseases including aquaporine-4-IgG-positive neuromyelitis optica spectrum disease (AQP4 + NMOSD) or myelin oligodendrocyte glycoprotein-IgG-associated disease (MOGAD) are reported.

**Methods:**

We performed a cross-sectional, retrospective spectral domain OCT study evaluating the frequency of PHOMS in AQP4 + NMOSD (*n* = 47) and MOGAD (*n* = 44) patients. To test the association with retinal neuroaxonal damage, we compared demographic and clinical data as well as retinal layer thicknesses between eyes with vs. eyes without PHOMS.

**Results:**

PHOMS were detected in 17% of AQP4 + NMOSD and 14% of MOGAD patients. Intra-cohort analysis revealed that AQP4 + NMOSD patients with PHOMS were significantly older [mean (years): 57.5 vs. 50.0; *p* value = 0.04]. We found no association of PHOMS with retinal neuroaxonal degeneration. In addition, in subjects with only one eye affected by PHOMS compared with the unaffected fellow eye, no differences in retinal parameters were observed (*n* = 4).

**Conclusions:**

In summary, we found PHOMS in 17% of AQP4 + NMOSD and 14% of MOGAD patients. This is comparable to the prevalence of published MS PHOMS data. Therefore, a disease-specific occurrence of PHOMS is unlikely. Interestingly, PHOMS do not seem to depend on retinal neuroaxonal degeneration.

## Introduction

Peripapillary hyper-reflective ovoid mass-like structures (PHOMS) are a recent finding in optical coherence tomography (OCT). PHOMS are thought to be associated with axoplasmic stasis and/or congestion in the glymphatic translaminar pressure system [1]. However, pathophysiology and significance of PHOMS remain unclear. Nevertheless, as a very new OCT marker, PHOMS have already been investigated in various neurological diseases including multiple sclerosis (MS). Here, a prevalence of PHOMS positivity was shown in 16–18% of patients [2, 3]. However, data on the prevalence of PHOMS in other inflammatory cerebral nervous system (CNS) disorders are lacking and little is known about the PHOMS frequency in adult healthy controls (HC). Therefore, we aimed to investigate the prevalence of PHOMS in patients with (i) aquaporine-4-IgG-positive (AQP4) neuromyelitis optica spectrum disorders (AQP4 + NMOSD) and (ii) myelin oligodendrocyte glycoprotein-IgG (MOG)-associated disease (MOGAD).

## Materials and methods

### Study design

In this cross-sectional, retrospective cohort study, patients with AQP4 + NMOSD and MOGAD were evaluated for the prevalence of PHOMS. All NMOSD and MOGAD patients from two university hospitals (Institute of Clinical Neuroimmunology, NeuroVisionLab, LMU Hospital; Department of Neurology, Klinikum rechts der Isar, TUM school of medicine, Technical University of Munich; recruitment: 2013 until 2021) with availability of clinical data and at least one OCT scan were included in the analysis. Diagnosis of AQP4 + NMOSD was defined by Wingerchuk et al. [4] and MOGAD by Jarius et al. [5]. Age- and sex-matched HC were included. Exclusion criteria were systemic or ophthalmologic diseases that might affect OCT data (arterial hypertension; diabetes mellitus; refraction error of > 5 dioptres; history of any known eye disease; eye surgery). All eyes with a (anamnestic and/or clinical) history of optic neuritis were excluded from OCT retinal layer analysis. The study was approved by the ethics committee of LMU and TUM and performed according to the Declaration of Helsinki. All individuals gave written consent.

### OCT imaging

All retinal scans were performed using a SPECTRALIS spectral domain (SD) OCT with automated eye tracking (OCT2-Module, Heidelberg Engineering as described before [3, 6]). Retinal layer segmentation was performed by the Heyex v2.5.5 (LMU) and Heyex v2.5.4 (TUM) software. The total macula volume (TMV), the combined ganglion cell and inner plexiform layer (GCIPL) and inner nuclear layer (INL) volume (all mm^3^) were acquired from 25 [30 × 25°, ART 13] vertical b-scans. The presence of PHOMS and the thickness (µm) of the peripapillary retinal nerve fibre layer (pRNFL) was assessed using a circular star-shaped optic disc scan centred on the optic nerve head (radial scan, 15° angle, 27 B-scans).

### Assessment of PHOMS

Radial scans were examined for presence of PHOMS according to the multirater consensus of 2020 [7] by two independent, experienced raters (RW; JAG) blinded to clinical information: PHOMS present (PHOMS +) vs. no PHOMS present (PHOMS−). Incoherent ratings were categorized as PHOMS + or PHOMS− in an open discussion (all authors).

### Statistical analysis

For statistical analysis, a paired eye approach was used to account for inter-eye correlations in each patient. For this purpose, mean values of both eyes were calculated if both eyes were available and assigned to the same group. GraphPad Prism (v9.1.1) was used for statistical analysis. Inter-rater agreement on rating of PHOMS per patient as well as eyes was calculated using Cohen´s kappa [8]. Patients with unilateral PHOMS were classified as PHOMS patients for further analysis. In case of unilateral PHOMS+, the PHOMS− eye was excluded from further analysis. For statistical analysis, we used the Fisher’s exact test for categorical data, the unpaired *t* test for normally distributed quantitative parameters and the Mann–Whitney *U* test for non-parametric data. Data are represented as median with corresponding 25–75% interquartile range. Statistical significance was set at *p* < 0.05.

## Results

### Study cohorts

In total, 131 subjects were screened, consisting of 81 AQP4 + NMOSD and 50 MOGAD patients. Of the patients screened, 47 AQP4 + NMOSD patients with 89 eyes, 44 MOGAD patients with 84 eyes met the stated inclusion criteria. Due to a previous ON, 35 eyes each had to be excluded in both the AQP4 + NMOSD (bilateral ON: *n* = 22 eyes, unilateral ON: *n* = 13 eyes) and MOGAD group (bilateral ON: *n* = 18 eyes, unilateral ON: *n* = 17 eyes) for further retinal layer analysis, but not for PHOMS analysis. A pool of 55 HC with 110 eyes was selected, of which 47 subjects could be analysed age- and sex-matched to the AQP4 + NMOSD cohort (HC.1) and 36 subjects to the MOGAD cohort (HC.2) (Tables 1, 2).

**Table 1:** Demographics and OCT characteristics in HC.1 and patients with AQP4 + NMOSD

	HC.1	AQP4 + NMOSD	*p*-value (HC.1 vs. AQP4 + NMOSD)
Demographics	*n* = 47, 94 eyes	*n* = 47, 89 eyes	
Sex female [number of patients (%)]	33 (70.2)	39 (83.0)	0.22
Age (years)	49.0 (40.0–56.0)	53.0 (43.0–59.0)	0.65
Disease duration (months)	n/a	57 (14–111)	n/a
EDSS	n/a	3 (2.5–4.0)	n/a
History of optic neuritis [number of patients (%)]	n/a	24 (51.0)	n/a
PHOMS [number of patients (%)]	2 (4.3)	8 (17.0)	0.09
OCT measurements	*n* = 47, 94 eyes	*n* = 35, 54 eyes	
pRNFL (µm)	100.0 (94.5–107.5)	99.5 (92.8–107.4)	0.70
TMV (mm^3^)	8.8 (8.5–9.0)	8.7 (8.4–8.9)	0.38
GCIPL (mm^3^)	2.1 (2.0–2.1)	2.0 (1.9–2.1)	0.30
INL (mm^3^)	0.96 (0.91–1.01)	0.97 (0.91–1.01)	0.76

AQP4+NMOSD		PHOMS +	PHOMS−	
Demographics		n = 8, 11 eyes	n = 39, 75 eyes	
Sex female [number of patients (%)]		5 (62.5)	34 (87.2)	0.12
Age (years)		57.5 (55.3–66.0)	50.0 (41.0–59.0)	**0.04**
Disease duration (months)		27.0 (9.3–93.8)	66.0 (14.0–128.0)	0.34
EDSS		3.5 (3.0–5.0)	3.0 (2.5–4.0)	0.36
History of optic neuritis [number of eyes (%)]		2 (18.2)	31 (41.3)	0.19
OCT measurements		*n* = 7, 9 eyes	n = 27, 44 eyes	
pRNFL (µm)		100.0 (93.5–112.0)	99.0 (92.0–106.0)	0.89
TMV (mm^3^)		8.5 (8.4–9.1)	8.6 (8.4–8.9)	0.86
GCIPL (mm^3^)		1.9 (1.9–2.1)	2.0 (1.9–2.1)	0.72
INL (mm^3^)		1.00 (0.86–1.07)	0.97 (0.92–1.01)	0.94

*AQP4* + *NMOSD* aquaporine-4-IgG-positive neuromyelitis optica spectrum disorders patients, *EDSS *Expanded Disability Scale,* GCIPL *ganglion cell-inner plexiform layer,* HC *healthy control,* INL *inner nuclear layer, *n *number of patients, *pRNFL *peripapillary retinal nerve fibre layer;* TMV *total macular volume

**Table 2:** Demographics and OCT characteristics in HC.2 and patients with MOGAD

	HC.2	MOGAD	*p*-value (HC.2 vs. MOGAD)
Demographics	*n* = 36, 72 eyes	*n* = 44, 84 eyes	
Sex female [number of patients (%)]	20 (52.8)	24 (54.6)	> 0.99
Age (years)	40.5 (29.3–51.8)	37.0 (28.3–44.8)	0.16
Disease duration (months)	n/a	15 (1–59.5)	n/a
EDSS	n/a	2.0 (1.5–4.0)	n/a
History of optic neuritis [number of patients (%)]	n/a	26 (59.0)	0.28
PHOMS [number of patients (%)]	2 (5.6)	6 (13.6)	n/a
OCT measurements	*n* = 36, 72 eyes	*n* = 33, 49 eyes	
pRNFL (µm)	101.5 (94.6–106.9)	100.0 (86.5–107.0)	0.18
TMV (mm^3^)	8.8 (8.6–9.1)	8.7 (8.4–8.9)	**0.03**
GCIPL (mm^3^)	2.1 (2.0–2.2)	2.0 (1.7–2.0)	**0.003**
INL (mm^3^)	0.96 (0.93–1.01)	0.96 (0.92–1.03)	0.70

MOGAD		PHOMS +	PHOMS−	
Demographics		*n* = 6, 7 eyes	*n* = 38, 72 eyes	
Sex female [number of patients (%)]		4 (66.7)	20 (52.6)	0.67
Age (years)		34.5 (24.8–41.3)	37.5 (28.8–47.0)	0.42
Disease duration (months)		34.5 (0.8–44.8)	8.0 (1.0–74.8)	0.74
EDSS		2.5 (1.4–4.6)	2.0 (1.5–3.5)	0.69
History of optic neuritis [number of eyes (%)]		1 (14.3)	32 (44.4)	0.23
OCT measurements		*n* = 5, 6 eyes	*n* = 38, 40 eyes	
pRNFL (µm)		92.0 (73.5–102.3)	100.0 (91.8–109.5)	0.27
TMV (mm^3^)		8.5 (7.7–8.8)	8.7 (8.4–8.9)	0.14
GCIPL (mm^3^)		1.9 (1.6–2.1)	2.0 (1.8–2.1)	0.70
INL (mm^3^)		0.99 (0.93–1.09)	0.96 (0.91–1.02)	0.50

*EDSS* Expanded Disability Scale, *GCIPL* ganglion cell-inner plexiform layer, *HC* healthy control, *INL* inner nuclear layer, *MOGAD* myelin oligodendrocyte glycoprotein-IgG associated disease patients, *n* number of patients, *pRNFL* peripapillary retinal nerve fibre layer, *TMV* total macular volume

### PHOMS rating

**Fig. 1:**
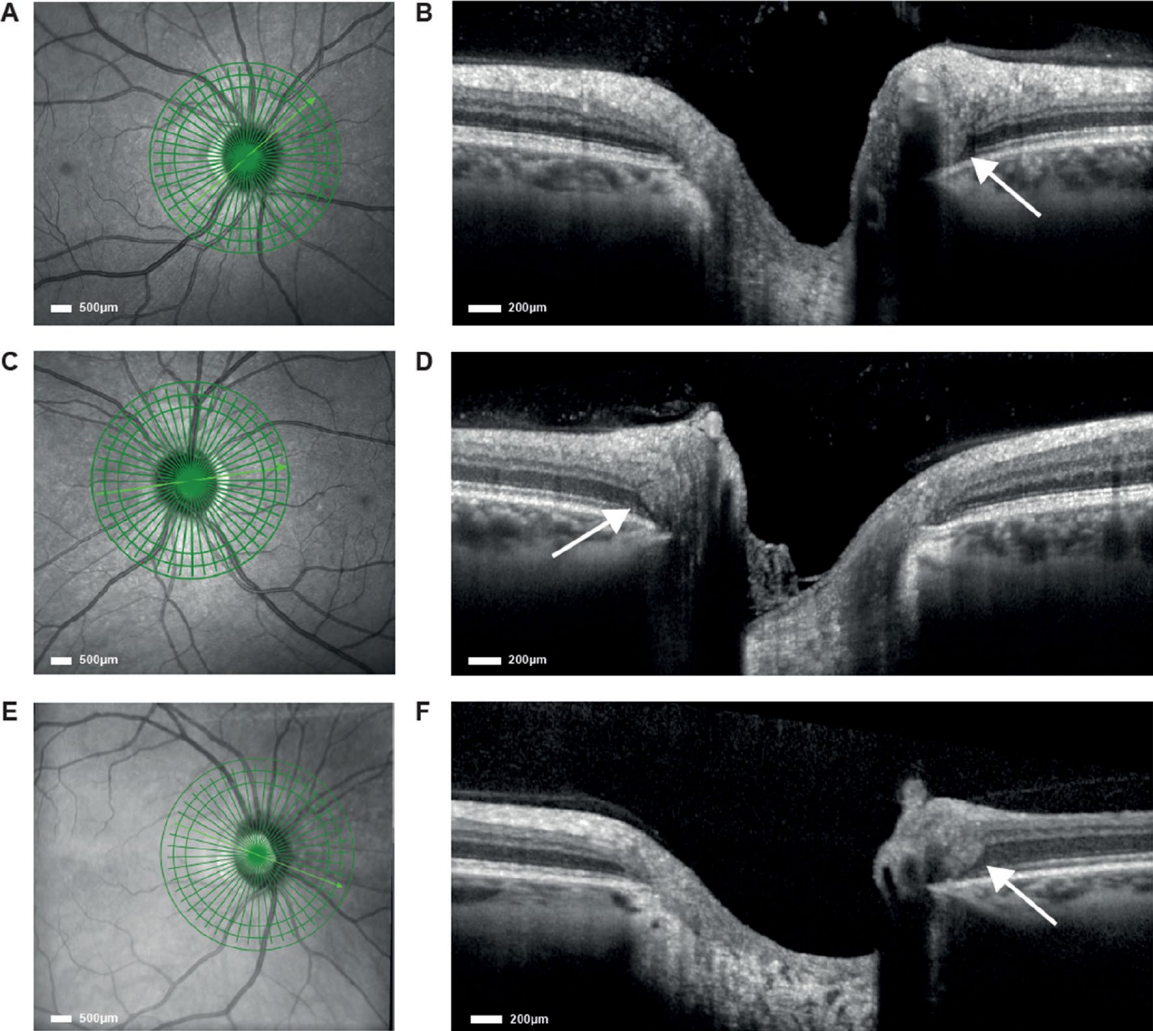
PHOMS detected in HC. **A** + **B** Right and **C** + **D** left eye of a 40-year-old man with detection of PHOMS in both eyes. **E** + **F** PHOMS + right eye of a 41-year-old woman without history of neurological disease

When evaluating all included eyes for occurrence of PHOMS an inter-rater agreement of 0.86 per patient (*κ* = 0.69, good inter-rater agreement), 0.91 per eye (*κ* = 0.55, moderate agreement) was reached. Radial scan images with PHOMS detected within the HC are presented in the Fig. 1.

### PHOMS in AQP4 + NMOSD

Within the AQP4 + NMOSD cohort, PHOMS were detected in 8 of 47 included patients (17% of patients; 11/89 [12%] eyes) and 2 healthy individuals of the age- and sex-matched HC cohort (4.3% of HC.1; 3/94 [3%] eyes). AQP4 + NMOSD PHOMS + patients were significantly older than PHOMS− patients (Table 1). No further clinical differences were observed between PHOMS + and PHOMS− patients with AQP4 + NMOSD or between NMOSD patients and its corresponding HC.1 cohort (Table 1). In eyes without optic neuritis (nON eyes), there was no significant difference in TMV, GCIPL, and INL, and pRNFL within the AQP4 + NMOSD cohort and compared with the HC.1 cohort.

### PHOMS in MOGAD

PHOMS were observed in 6 of 44 included MOGAD patients (14% of patients; 7/84 [8%] eyes) and 2 healthy individuals of the age- and sex-matched HC cohort (5.6% of HC.2; 3/72 [4%] eyes). No significant clinical or demographic differences could separate PHOMS + from PHOMS− patients. There was no association between the presence of PHOMS and retinal neuroaxonal damage (Table 2). However, TMV and GCIPL volumes of nON eyes were reduced in MOGAD patients compared with HC.2. Within the corresponding HC.2 cohort, the same 2 individuals with PHOMS were detected as in HC.1 cohort related to the AQP4 + NMOSD cohort (Fig. 1).

### OCT characteristics in subjects with unilateral PHOMS

A total of 4 individuals (25% of all PHOMS + subjects) showed PHOMS detection unilaterally only (1 AQP4 + NMOSD, 2 MOGAD, 1 HC). This cohort also showed no significant difference in retinal neuroaxonal degeneration of the PHOMS + eye compared with the PHOMS- eye (data not shown).

## Discussion

We found PHOMS in 17% of patients with AQP4 + NMOSD and in 14% with MOGAD. In both cohorts, the proportion of patients with PHOMS was comparable to previously published data on PHOMS prevalence in MS [2, 3]. Occurrence of PHOMS in AQP4 + NMOSD and MOGAD patients was not associated with sex, disease duration, disability and retinal neuroaxonal degeneration. Previous studies have shown that retinal neuroaxonal degeneration is prominent in nON eyes in MS patients, whereas ON-independent retinal neuroaxonal degeneration in AQP4 + NMOSD and MOGAD currently remains under debate [9–11]. If PHOMS occur in relation to the extent of neuroaxonal degeneration, a different prevalence between the diseases could be suspected. Based on our data, no association between retinal neuroaxonal degeneration and PHOMS prevalence is apparent. Interestingly, the only factor we found was an age-dependent association of PHOMS prevalence in AQP4 + NMOSD patients.

In addition to a possible association with retinal neuroaxonal degeneration, it is speculated that an impaired retinal glymphatic system or axoplasmatic congestion might be linked to PHOMS [2]. The glympathic system has been studied in the context of neurodegenerative diseases [12], whereas little is known in MS, NMOSD or MOGAD [13]. One could speculate that there is a direct link to the glymphatic system in AQP4 + NMOSD through the presence of AQP4-IgG. The functional integrity of aquaporin-4 water channels is essential for retinal homeostasis [12, 14]. Binding of AQP4-IgG might disrupt the retinal glymphatic system and thus lead to an increased frequency of PHOMS in AQP4 + NMOSD [14]. In addition, the age-dependent increase in PHOMS frequency within our AQP4 + NMOSD cohort might support the hypothesis that PHOMS could develop de novo resulting from an age-dependent impairment of the glymphatic outflow. However, we found a comparable prevalence of PHOMS in AQP4 + NMOSD compared with MOGAD and published MS data making the above-mentioned hypothesis unlikely [2, 3]. To close the knowledge gap, histopathological studies of PHOMS eyes are necessary for detailed disease understanding.

Finally, it is unclear whether PHOMS only evolve under disease conditions or whether they also appear under healthy states. Here, little is known about the prevalence of PHOMS in HCs: (i) Petzold et al. reported a HC group containing 59 subjects without any PHOMS detected [2]; (ii) Hamann et al. described 53 patients with unilateral non-arteritic anterior ischaemic optic neuropathy (NA-AION) and found 5 PHOMS in 30 unaffected (no NA-AION) and optic drusen-free eyes [15]. We found evidence of PHOMS in 4% of HCs. However, for our PHOMS + HCs, we cannot definitively exclude a possible subclinical ophthalmologic, neurologic, or vascular disease, because the information on ophthalmologic comorbidities was based solely on the medical history of the included HCs. Thus, it is necessary to independently investigate the prevalence of PHOMS in larger HC cohorts. Nevertheless, our data show a higher prevalence of PHOMS in AQP4 + NMOSD and MOGAD, suggesting a general disease dependency of the occurrence of PHOMS.

The power of our study is limited due to the retrospective cross-sectional nature, the sample sizes of very rare diseases and the ON classification mainly based on anamnestic information. An international, multicentre design with longitudinal assessment of PHOMS could provide further clarity on this issue.

## Conclusions

In summary, we found PHOMS in 17% of AQP4 + NMOSD and 14% of MOGAD patients comparable to published MS PHOMS data. A disease-specific occurrence is unlikely, but a disease-dependent occurrence can be assumed with a higher prevalence of PHOMS compared to HCs. Therefore, a dedicated examination for PHOMS in different disease groups is useful, especially since the presence of PHOMS could also be mistakenly classified as papilledema in the ophthalmological examination.
